# Supplementary material for: Putting the Personalized Metabolic Avatar into Production: A Comparison between Deep-Learning and Statistical Models for Weight Prediction
Source: Nutrients. 2023 Feb 27;15(5):1199. doi: 10.3390/nu15051199 (PMC10004838; doi:10.3390/nu15051199)
Supplement: Supplementary file 1 [file nutrients-15-01199-s001.zip › nutrients-2239438-supplementary.pdf]

nutrients

MDPI

Supplementary Materials

Section S1. SARIMAX architecture

We start from a time series wt. An AR(p) model can be specified by

$$w_t = \beta + \epsilon_t + \sum_{i=1}^{p} \theta_i w_{t-1}, \quad \text{(S1)}$$

Where p is the number of time lags to regress on, $\boldsymbol{\epsilon_t}$ is the noise at time t and β is a constant. This equation can be made more concise using the lag operator, L.

$$L^n w_t = w_{t-n}, \quad \text{(S2)}$$

Taking $\boldsymbol{\Theta}$ (L) to be an order p polynomial function of L, we can define an autoregressive model by

$$y_t = \Theta(L)^p y_t + \epsilon_t, \quad \text{(S3)}$$

Taking note that the constant has been absorbed into the polynomial $\boldsymbol{\Theta}$. Whereas autoregressive models regress on prior values of wt, moving average models regress on prior values of error. An $MA(q)$ model can be specified by

$$w_t = \Phi(L)^q \epsilon_t + \epsilon_t, \quad \text{(S4)}$$

where q is the number of time lags of the error term to regress on and $\boldsymbol{\Phi}$ is defined analogously to $\boldsymbol{\Theta}$. $ARMA(p,q)$ models are a sum of $AR(p)$ and $MA(q)$ models.

$$w_t = \Theta(L)^p w_t + \Phi(L)^q \epsilon_t + \epsilon_t w_t, \quad \text{(S5)}$$

To help tackle non-stationary data, we introduce an integration operator $\Delta^{\boldsymbol{d}}$, defined as follows

$$\Delta^d w_t = w_t^{[d-1]} - w_{t-1}^{[d-1]}, \quad \text{(S6)}$$

where $\Delta^{\boldsymbol{0}} \boldsymbol{w_t} = \boldsymbol{w_t}$ and d is the order of differencing used.

We can now fit an $ARMA(p,q)$ model to $\Delta^{\boldsymbol{d}} \boldsymbol{w_t}$

$$\Delta^d w_t = \Theta(L)^p \Delta^d w_t + \Phi(L)^q \Delta^d \epsilon_t + \Delta^d \epsilon_t, \quad \text{(S7)}$$

With some algebra, we can rearrange the equation and absorb constants into the polynomials $\boldsymbol{\Theta}$ and $\boldsymbol{\Phi}$ obtaining

$$\Theta(L)^p \Delta^d w_t = \Phi(L)^q \Delta^d \epsilon_t, \quad \text{(S8)}$$

SARIMA models take seasonality into account by essentially applying an ARIMA model to lags that are integer multiples of seasonality. Once the seasonality is modeled, an ARIMA model is applied to the leftover to capture non-seasonal structure. To see this more clearly, suppose we have a time series wt with seasonality s. We can try to eliminate the seasonality with differencing, by applying the differencing operator $\Delta_{\boldsymbol{s}}^{\boldsymbol{D}}$ to take the seasonal differences of the time series. Here, s is the number of time lags comprising one full period of seasonality. D takes on a similar meaning to d in ARIMA models, but instead applies to seasonal lags. We can then capture any remaining structure by applying an $ARMA(P,Q)$ model, to the different values, but using seasonal lags. i.e., instead of using a regular lag operator L, we use $\boldsymbol{L^s}$. P and Q are again seasonal time lags

$$\Delta_s^D w_t = \theta(L^s)^P \Delta_s^D w_t + \varphi(L^s)^Q \Delta_s^D \epsilon_t + \Delta_s^D \epsilon_t, \quad \text{(S9)}$$

As with ARIMA, massaging the equation and absorbing constants into polynomials yields the following concise form

$$\theta(L^s)^P \Delta_s^D w_t = \varphi(L^s)^Q \Delta_s^D \epsilon_t, \quad \text{(S10)}$$

With any seasonality now removed, we can apply another $ARIMA(p,d,q)$ model to sDwt by multiplying the seasonal model by the new ARIMA model.

$$\Theta(L)^p \theta(L^s)^P \Delta^d \Delta_s^D w_t = \Phi(L)^q \varphi(L^s)^Q \Delta^d \Delta_s^D \epsilon_t, \quad \text{(S11)}$$

This is the general form of a $SARIMA(p,d,q)(P,D,Q,s)$ model.

SARIMAX models take exogenous variables into account - i.e., variables measured at time t that influence the value of our time series at time t, but that are not autoregressed on. To do this, we simply add the terms in on the right-hand side of SARIMA equations.

For n exogenous variables defined at each time step t, denoted by $\boldsymbol{x_t^i}$ for $i \leqslant n$ with coefficients $\boldsymbol{\beta_i}$, the $SARIMAX(p,d,q)(P,D,Q,s)$ model is

$$\Theta(L)^p \theta(L^s)^p \Delta^d \Delta_s^D w_t = \Phi(L)^q \varphi(L^s)^Q \Delta^d \Delta_s^D \epsilon_t + \sum_{i=1}^{n} \beta_i x_t^i. \quad \text{(S12)}$$

Section S2. Transformer architecture

The Transformer [1] follows the encoder-decoder architecture, quite common in most competitive neural sequence transduction models [2–5], using stacked self-attention and pointwise, fully connected layers for both encoder and decoder (Figure S1).

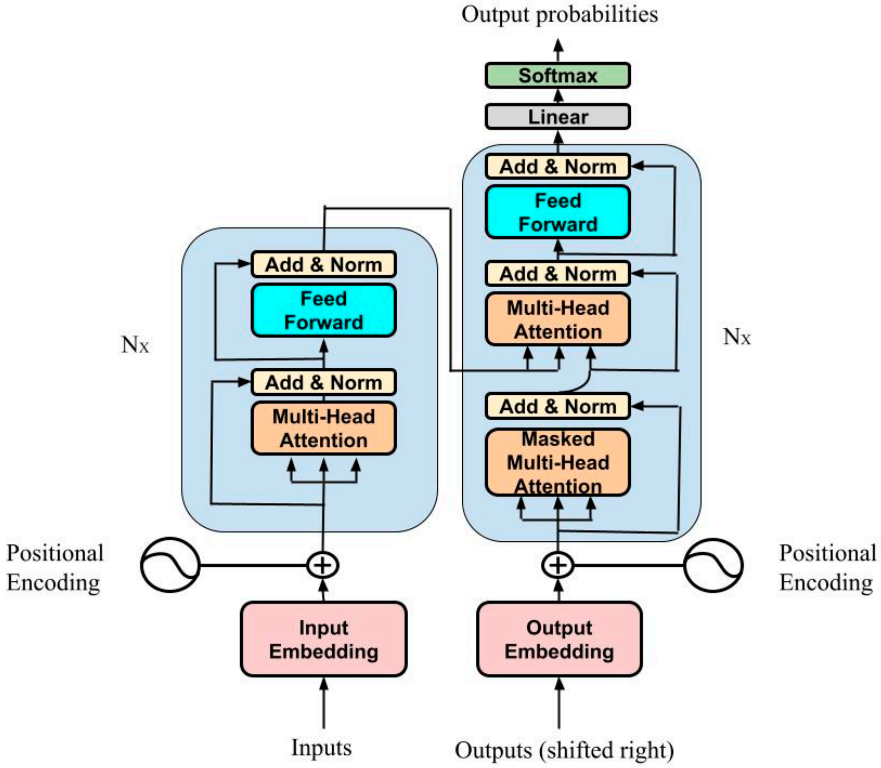

Figure S1. Schema representing Transformer architecture.

In particular, the encoder and decoder are composed of a stack of N = 6 identical layers that contain two sublayers each one (a multi-headed self-attention mechanism and a simple position wise fully connected feed-forward network) around which a residual connection is used, followed by layer normalization. In the decoder, in addition, there is also a third layer which performs multi-head attention over the output of the encoder stack, that can be resume as:

$$LayerNorm(x + Sublayer(x)), \quad \text{(S13)}$$

where Sublayer(x) is the function implemented by the sublayer itself.

For the decoder, the self-attention sub-layer is also modified to prevent positions from attending to subsequent positions. This masking, combined with the fact that the output embeddings are offset by one position, ensures that the predictions for position i can depend only on the known outputs at positions less than i.

In this configuration, the attention functions, mapping a query and a set of key-value pairs to an output (the query, keys, values and output are all vectors), compute the output as a weighted sum of the values, where these weights are computed by a compatibility function of the query with the corresponding key.

For the Transformer's architecture the Scaled Dot-Product Attention function is used. In this function, the input consists of queries and keys of dimension $\boldsymbol{d_k}$, and values of dimension $\boldsymbol{d_v}$.

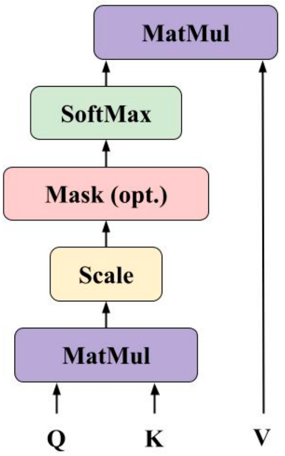

Figure S2. Scaled Dot-Product Attention schematic.

This block (Figure S2) computes the dot products of the query with all keys, divided each by $\sqrt{d_k}$, and applies a softmax function to obtain the weights on the values. In practice, the attention function is computed on a set of queries simultaneously, packed together into a matrix Q. The keys and values are also packed together into matrices K and V. The matrix of outputs is computed as:

$$L^n Attention(Q,K,V) = softmax\left(\frac{QKT}{\sqrt{d_k}}\right)V, \quad (S14)$$

The Scaled Dot-Product Attention is an evolution of the Dot-Product Attention, where the scaling of $\frac{1}{\sqrt{d_k}}$ allows to counteract a problem for large values of d_k, where the dot products could grow large in magnitude and push the softmax function into regions where it has extremely small gradients.

However, with a single attention head, averaging inhibits the model to jointly attend to information from different representation subspaces at different positions. Therefore, instead of performing a single attention function with d_{model}-dimensional keys, values and queries, it is advantageous to linearly project the queries, keys and values h times with different, learned linear projections to d_k, d_k and d_v dimensions, respectively. On each of these projected versions of queries, keys and values then the attention function is performed in parallel, yielding d_v-dimensional output values. These are concatenated and once again projected, resulting in the final values, as depicted in Figure S3.

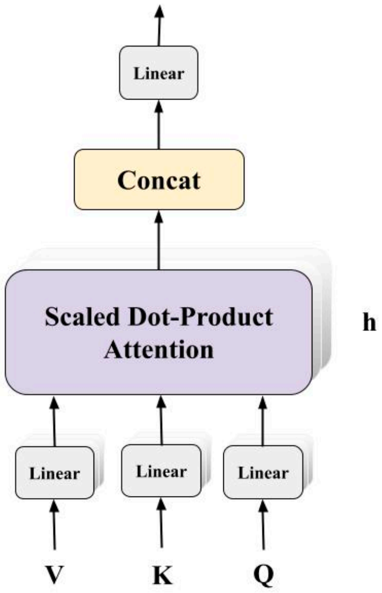

Figure S3. Multi-Head Attention schematic.

This is called Multi-Head Attention and it could be represented through the following formula:

$$MultiHead(Q,K,V) = Concat(head,\dots,head_n)W^O, \quad \text{(S15)}$$

where $\boldsymbol{head_i} = \boldsymbol{Attention}(\boldsymbol{QW_i^Q}, \boldsymbol{KW_i^K}, \boldsymbol{VW_i^V})$ and the projections are parameter matrices $\boldsymbol{W_i^Q} \in \mathbb{R}^{d_{model} \times d_k}$, $\boldsymbol{W_i^K} \in \mathbb{R}^{d_{model} \times d_k}$, $\boldsymbol{W_i^V} \in \mathbb{R}^{d_{model} \times d_v}$ and $\boldsymbol{W_i^O} \in \mathbb{R}^{hd_k \times d_{model}}$.

For example, considering h=8 parallel attention layers in the tuning of the model, or heads, for each of them then $\boldsymbol{d_k} = \boldsymbol{d_v} = \frac{\boldsymbol{d_{model}}}{\boldsymbol{h}} = \boldsymbol{64}$. Due to the reduced dimension of each head, the total computational cost is like that of single-head attention with full dimensionality.

The Transformer uses the Multi-Head Attention in three different ways:

In the decoder we have two types of attention:

- The first one, the self-attention layer, contains self-attention layers where all the keys (K), values (V) and queries (Q) come from the same place (the output of the previous layer). In such a way, each position in the decoder can attend to all positions in the previous layer. This applies also for the self-attention layer of the encoder.
- Instead, in the second one, the encoder-decoder attention layers, Q come from the previous layer as well, but K and V come from the output of the encoder allowing every position in the decoder to attend over all positions in the input sequence, mimicking the typical encoder-decoder attention mechanisms in sequence-to-sequence models [2,6,7].

As explained earlier, in addition to attention sub-layers, the encoder and decoder layers also contain a fully connected feed-forward network, which is applied to each position separately and identically, with two linear transformations alternating with ReLU activation in between.

$$FFN(x) = \max(0, xW_1 + b_1)\, W_2 + b_2, \quad \text{(S16)}$$

Despite being the same across different layers, linear transformations use different parameters.

Transformer uses sine and cosine function of different frequencies as positional encoding to the input embedding at the bottoms of the encoder and decoder stacks to add some information about the relative or absolute position of the tokens in the sequence.

$$PE_{(pos,2i)} = sin\,\left(\frac{pos}{10000^{\frac{2i}{d_{model}}}}\right), \quad \text{(S17)}$$

$$PE_{(pos,2i+1)} = cos\,\left(\frac{pos}{10000^{\frac{2i}{d_{model}}}}\right), \quad \text{(S18)}$$

where *pos* is the position and i is the dimension. In this way, each dimension of the positional encoding corresponds to a sinusoid. The wavelengths form a geometric progression from 2π to 10000 · 2π. This function allows the model to easily learn to attend to relative positions, since for any fixed offset k, $\boldsymbol{PE_{pos+k}}$ can be represented as a linear function of $\boldsymbol{PE_{pos}}$.

Section S3. Models' parameters

Table S1. The results of the tuning of each model are reported in the table, in particular for the SARIMAX model the parameters are the following: (p,dq)x(P,D,Q,S); for the LSTM and GRU: (units, epochs, batch size, dropout); for the Transformer: (head size, num heads, epochs, batch size, dropout).

Users	SARIMAX	LSTM	GRU	Transformer
0	(4,1,2)x(1,1,1,7)	(100,150,16,0.2)	(50,100,8,0.2)	(256,2,100,8,0.25)
1	(2,1,1)x(1,1,1,7)	(150,50,16,0.2)	(100,100,32,0.2)	(128,8,50,8,0.25)
2	(2,1,1)x(1,1,1,7)	(50,150,32,0.2)	(100,50,32,0.2)	(256,2,50,8,0.25)
3	(2,1,1)x(1,1,1,7)	(50,100,32,0.2)	(100,100,32,0.2)	(64,2,50,8,0.2)
4	(2,2,2)x(1,1,1,7)	(100,50,16,0.2)	(150,50,16,0.2)	(128,2,50,32,0.2)

5	(2,1,2)x(1,1,1,7)	(100,100,16,0.2)	(150,150,8,0.2)	(64,2,50,16,0.2)
6	(1,1,2)x(1,1,1,7)	(150,150,8,0.2)	(100,50,8,0.2)	(256,2,50,16,0.2)
7	(2,1,1)x(1,1,1,7)	(150,50,16,0.2)	(150,100,16,0.2)	(64,2,150,32,0.2)
8	(1,2,2)x(1,1,1,7)	(100,100,16,0.2)	(100,50,16,0.2)	(64,4,100,16,0.2)
9	(3,1,1)x(1,1,1,7)	(150,150,8,0.2)	(50,150,32,0.2)	(128,8,100,8,0.2)

References

1. Vaswani, A.; Brain, G.; Shazeer, N.; Parmar, N.; Uszkoreit, J.; Jones, L.; Gomez, A.N.; Kaiser, Ł.; Polosukhin, I. *Attention Is All You Need*; 2017;
2. Bahdanau, D.; Cho, K.; Bengio, Y. Neural Machine Translation by Jointly Learning to Align and Translate. **2014**.
3. Sutskever Google, I.; Vinyals Google, O.; le Google, Q. v *Sequence to Sequence Learning with Neural Networks*;
4. Cho, K.; van Merrienboer, B.; Gulcehre, C.; Bahdanau, D.; Bougares, F.; Schwenk, H.; Bengio, Y. Learning Phrase Representations Using RNN Encoder-Decoder for Statistical Machine Translation. **2014**.
5. Kim, Y.; Denton, C.; Hoang, L.; Rush, A.M. Structured Attention Networks. **2017**.
6. Gehring, J.; Auli, M.; Grangier, D.; Yarats, D.; Dauphin, Y.N. Convolutional Sequence to Sequence Learning. **2017**.
7. Wu, Y.; Schuster, M.; Chen, Z.; Le, Q. v.; Norouzi, M.; Macherey, W.; Krikun, M.; Cao, Y.; Gao, Q.; Macherey, K.; et al. Google's Neural Machine Translation System: Bridging the Gap between Human and Machine Translation. **2016**.

Disclaimer/Publisher's Note: The statements, opinions and data contained in all publications are solely those of the individual author(s) and contributor(s) and not of MDPI and/or the editor(s). MDPI and/or the editor(s) disclaim responsibility for any injury to people or property resulting from any ideas, methods, instructions or products referred to in the content.
